# *Hassalstrongylus dollfusi* (Nematoda, Heligmonellidae): rediscovery in native South American rodents, six decades after its description

**DOI:** 10.1051/parasite/2021077

**Published:** 2021-12-09

**Authors:** Paula Carolina Serrano, María Celina Digiani, María de los Angeles Gómez-Muñoz, Juliana Notarnicola, María del Rosario Robles, Graciela Teresa Navone

**Affiliations:** 1 CONICET-Consejo Nacional de Investigaciones Científicas y Técnicas Argentina; 2 División Zoología Invertebrados, Facultad de Ciencias Naturales y Museo, Universidad Nacional de La Plata Paseo del Bosque s/n 1900 La Plata Argentina; 3 Laboratorio Biología de los Parásitos, Facultad de Ciencias Exactas y Naturales y Agrimensura, Universidad Nacional del Nordeste Av. Libertad 5460 3400 Corrientes Argentina; 4 Instituto de Biología Subtropical (CONICET - Universidad Nacional de Misiones) Bertoni 85 3370 Puerto Iguazú Argentina; 5 Centro de Estudios Parasitológicos y de Vectores (CCT La Plata-CONICET-UNLP), 120 e/61 y 62 B1900FWA La Plata Argentina

**Keywords:** Nippostrongylinae, Helminths, Sigmodontinae, Primary hosts, Northeast Argentina, Venezuela

## Abstract

*Hassalstrongylus dollfusi* (Díaz-Ungría, 1963) Durette-Desset, 1971 was described in a wild house mouse, *Mus musculus*, from Venezuela and, since then, has never been reported again in the type host or in any other host. In this work, specimens assignable to *H. dollfusi* were found at 10 localities in Northeast Argentina, in five species of sigmodontine rodents. The nematodes were attributed to *H. dollfusi* based on diagnostic characters such as: synlophe with 22–31 subequal ridges; in males, hypertrophy of right ray 4 of the male bursa, thickening of the dorsal ray and bases of rays 8, distal tip of the spicules bent and spoon shaped; and, in females, presence of subventral postvulvar alae supported by hypertrophied struts. The new host recorded are: *Oligoryzomys fornesi*, *O. flavescens*, *O. nigripes*, *Holochilus chacarius* and *Akodon azarae*. The parasite showed a strong preference for host species of *Oligoryzomys*, which appear to act as primary hosts. The parasite could be present, parasitizing different species of *Oligoryzomys*, in a geographic area from the type locality in Venezuela southward to north Corrientes in Argentina. It has not been reported from populations of *Oligoryzomys* spp. of the Argentinean and Brazilian Atlantic Forest, nor south of 28° S, which may be explained by constraints in the environmental conditions required by the free-living stages of the parasite. This study provides the first identification and redescription of *H. dollfusi* in southern South America, from autochthonous hosts, six decades after its description.

## Abstract (fr)

*Hassalstrongylus dollfusi* (Díaz-Ungría, 1963) Durette-Desset, 1971 a été décrit chez une souris grise sauvage, *Mus musculus*, au Venezuela et, depuis lors, n’a plus jamais été signalé chez l’hôte-type ni chez aucun autre hôte. Dans ce travail, des spécimens attribuables à *H. dollfusi* ont été trouvés dans dix localités du nord-est de l’Argentine, chez cinq espèces de rongeurs Sigmodontinae. Les nématodes ont été attribués à *H. dollfusi* sur la base de caractères diagnostiques tels que : synlophe avec 22–31 crêtes subégales; chez le mâle, hypertrophie de la côte 4 droite de la bourse, épaississement de la côte dorsale et des bases des côtes 8, extrémité distale des spicules recourbée et en forme de cuillère; et, chez les femelles, présence d’ailes postvulvaires subventrales à fort support cuticulaire. Les nouveaux hôtes signalés sont : *Oligoryzomys fornesi*, *O. flavescens*, *O. nigripes*, *Holochilus chacarius* et *Akodon azarae*. Le parasite a montré une forte préférence pour les espèces hôtes du genre *Oligoryzomys*, qui semblent agir comme hôtes primaires. Le parasite pourrait être présent, parasitant différentes espèces d’*Oligoryzomys*, dans une zone géographique allant de la localité-type au Vénézuéla vers le sud jusqu’au nord de Corrientes en Argentine. Il n’a pas été signalé dans les populations d’*Oligoryzomys* spp. de la Forêt Atlantique argentine et brésilienne, ni au sud de 28° S, ce qui peut s’expliquer par des contraintes dans les conditions environnementales requises par les stades libres du parasite. Cette étude fournit la première identification et redescription d’*H. dollfusi* dans le sud de l’Amérique du Sud, à partir d’hôtes autochtones, six décennies après sa description.

## Introduction

The Heligmonellidae are the most speciose family of the Trichostrongylina (Strongylida or bursate nematodes). Heligmonellids are distributed worldwide and are overwhelmingly parasites of rodents (ca. 350 species); very few species have been described from moles, lagomorphs, tragulids and dermopterans (< 20) [3, 22]. Among the five subfamilies of heligmonellids, the Nippostrongylinae include the largest number of species (ca. 230); nippostrongylines are widespread all over the world and occur mainly in the superfamily Muroidea, frequently with more than one species per host [22].

In the Americas, nippostrongylines are parasitic in the Cricetidae (Muroidea), which are represented by four subfamilies: the Arvicolinae (with Holarctic distribution), the Neotominae and Tylomyinae (North or Middle American lineages); and the highly diverse Sigmodontinae (autochthonous to South America) [36]. Together, these subfamilies include ca. 650 species of rodents [43]. In contrast, less than 10% of these host species (ca. 60) have been investigated for nippostrongylines, which are known to be a highly diverse group [13].

In addition to native cricetids, synanthropic species of rats and mice: *Mus musculus* Linnaeus, *Rattus norvegicus* Berkenhout and *Rattus rattus* Linnaeus (Muridae, Murinae) also act as hosts to nippostrongylines. Due to their importance in public health, the parasitic fauna of these rodents has been much more extensively surveyed ([1, 11, 24–27, 30, 34, 35] among the most recent). Both species of *Rattus* mentioned above harbour throughout their distribution their own species of nippostrongyline: *Nippostrongylus brasiliensis* (Travassos, 1914) [9, 40]. However, they can occasionally be parasitized with other nippostrongylines from autochthonous rodents [14, 20, 35]. In a similar way, domestic mice, though they are not usually parasitized by nippostrongylines, may become occasionally infected with *N. brasiliensis* [9, 34] and with nippostrongylines from native rodents [12, 14, 23, 35].

Among New World nippostrongylines, the genus *Hassalstrongylus* Durette-Desset, 1971 [16] includes 17 species distributed from the southern USA to central Argentina. Fourteen out of the 17 species parasitize only native sigmodontine hosts; however, *Hassalstrongylus aduncus* (Chandler, 1932), primarily parasitic in Sigmodontinae and Arvicolinae [4, 10, 17, 23], has also been reported in rats [20, 35] and *Hassalstrongylus musculi* (Dikmans, 1935), although described originally in mice [14], was later found repeatedly parasitizing several species of sigmodontines [29, 35, 41].

*Hassalstrongylus dollfusi* (Díaz-Ungría, 1963) was originally described as *Longistriata dollfusi* Díaz-Ungría, 1963 in a wild *Mus musculus* from Venezuela [12]. Some years later, Durette-Desset (1969) [15] described the synlophe on re-examination of the type material, and finally Durette-Desset (1971) [16] transferred the species to the genus *Hassalstrongylus*. These three contributions are the only existing publications concerning *H. dollfusi*, there having been no new records since its description in 1963. It was, up to now, the only species of *Hassalstrongylus* reported exclusively from an exotic Murinae.

In this work, we report the finding of *H. dollfusi* in five species of sigmodontine hosts in 10 different localities of Northeast Argentina, providing a complete morphological description and enlarging the range of morphometrical data on this species. Additionally, we provide ecological parameters for the different surveyed populations and discuss the status of the different host-parasite associations.

## Materials and methods

### Ethics

The research was performed in agreement with Argentine laws. The specimens, obtained using methods for live capture, were sacrificed following the procedures and protocols suggested by AVMA Guidelines on Euthanasia and approved by National Laws (Animal Protection National Law 14.346 and references in the provincial permits) and by the Ethics Committee for Research on Laboratory Animals, Farm and Obtained from Nature of the National Council of Scientific and Technical Research (CONICET). No endangered species were involved in this study.

### Materials

**Figure 1:**
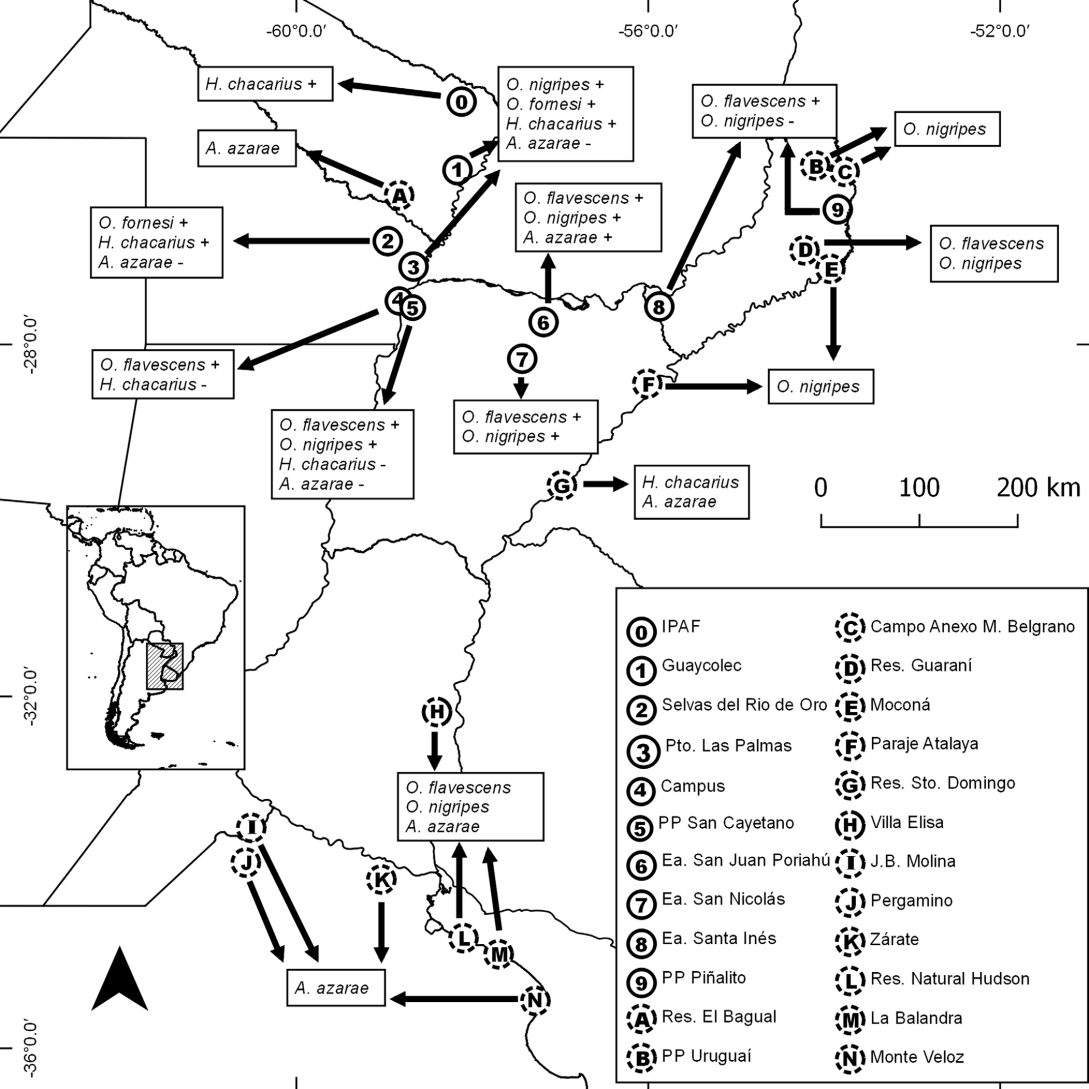
Sampled localities, including presence (0–9) and absence (A–N) of *Hassalstrongylus dollfusi*. Plus and minus symbols indicate the presence and absence of the parasite in each host, respectively. Localities B and C taken from Panisse [32]; Localities L and M taken from Navone et al. [31].

In the framework of a more comprehensive project involving the study of the taxonomy, host and geographical distribution of different groups of helminths of sigmodontine rodents in Argentina, viscera of 580 rodents belonging to 13 species were examined for helminths: *Akodon azarae* (J. B. Fischer), *n* = 106; *Akodon montensis* Thomas, *n* = 94; *Calomys callidus* (Thomas), *n* = 4; *Calomys callosus* (Rengger), *n* = 16; *Holochilus chacarius* Thomas, *n* = 50; *Holochilus vulpinus* (Brants), *n* = 9; *Necromys lasiurus* (Lund), *n* = 77; *Necromys obscurus* (Waterhouse), *n* = 10; *Oligoryzomys flavescens* (Waterhouse), *n* = 38; *Oligoryzomys fornesi* (Massoia), *n* = 14; *Oligoryzomys nigripes* (Olfers), *n* = 99; *Oxymycterus rufus* (G. Fischer), *n* = 56 and *Sooretamys angouya* (G. Fischer), *n* = 7. Rodents were captured during several field studies involving various collaborators (see Acknowledgements) between 1999 and 2018 from a total of 29 localities distributed in seven provinces. The results presented herein refer only to the rodent species harbouring *H. dollfusi* (Fig. 1).

### Methods

Nematodes were recovered by observation of the gastrointestinal tracts under a stereomicroscope, subsequently fixed in 4% formalin and preserved in 70% ethanol. Nippostrongylines were studied in temporary mounts in Amman’s lactophenol under a Leica DM 2500 microscope provided with a drawing attachment. Photographs were taken with a Leica DMC5400 camera. The synlophe was studied following Durette-Desset [18] and the nomenclature referring to the axis of orientation of the ridges follows Durette-Desset and Digiani [19]. Ridges are numbered from left to right and with respect to the oblique axis of orientation: 1 to n for right-dorsal ridges, and 1′ to n′ for left-ventral ridges. The nomenclature used for the study of the bursa (pattern of lateral lobes and symmetry) follows Durette-Desset et al. [21]. Measurements, unless otherwise stated, are provided in micrometres as the range followed by the mean in parentheses. SpL/BL and UtL/BL mean the proportion of the spicule length to the body length and of the uterus length to the body length, respectively. EP/OeL means the position of the excretory pore (in percentage) with respect to the oesophagus length. Ecological parameters (prevalence and mean intensity) were calculated according to Bush et al. [5]. With the aim of evaluating the status of the different hosts harbouring *H. dollfusi*, the relative dominance (RD) of the latter in relation to the other intestinal heligmonellid species was calculated for each host species and locality. Figure 1 was constructed with free and open code software QGIS 2.14 [37].

Voucher specimens of helminths were deposited in the Helminthological Collection of the Museo de La Plata, La Plata, Buenos Aires, Argentina (MLP-He). Voucher specimens of hosts were deposited in the Mammalogical Collection of the Museo de La Plata (MLP-Mz) and Mammalogical Collection of the Centro Nacional Patagónico–CENPAT-CCT-CONICET, Puerto Madryn, Chubut, Argentina (CNP).

## Results

### *Hassalstrongylus dollfusi* (Díaz-Ungría, 1963) Durette-Desset, 1971

(= *Longistriata dollfusi* Díaz-Ungría, 1963)

*Site of Infection:* Small intestine.

*New hosts recorded: Akodon azarae* (Sigmodontinae, Akodontini), *Holochilus chacarius*, *Oligoryzomys flavescens*, *O. fornesi*, *O. nigripes* (Sigmodontinae, Oryzomyini).

*Localities* (Fig. 1, Table 1):

(0) Instituto de Investigación para la Pequeña Agricultura Familiar del Noreste Argentino (IPAF- NEA), Laguna Blanca, Departamento Pilcomayo, Formosa province (25°12′09.91″ S, 58°07′13.71″ W) (IPAF) (only *H. chacarius* examined from this locality. Not included in Table 1);
(1) Estación de Animales Silvestres Guaycolec, Departamento Formosa, Formosa province (25°58′57.08″ S, 58°10′04.00″ W) (GUAY);
(2) Selvas del Río de Oro, Departamento Libertador General San Martín, Chaco province, (26°47′23.35″ S, 58°57′37.81″ W) (SRDO)
(3) Puerto Las Palmas, Departamento Bermejo, Chaco province, three sampling points (27°04′45.00″ S, 58°40′06.30″ W); 7 km S Puerto Las Palmas (27°09′40.51″ S, 58°40′27.29″ W); 2.5 km NW Puerto Las Palmas (27°04′45.00″ ′S, 58°40′06.30″ W) (PULP);
(4) Campus “Deodoro Roca”-Universidad Nacional del Nordeste, Departamento Capital, Corrientes province (27°28′09.17″ S, 58°49′50.29″ W) (CAMP);
(5) Parque Provincial San Cayetano, Departamento Capital, Corrientes province, two sampling points (27°32′43.88″ S, 58°40′33.73″ W); (27°34′15″ S; 58°41′41″ W) (SCAY);
(6) Estancia San Juan Poriahú, Departamento San Miguel, Corrientes province (27°42′34.09″ S, 57°11′19.08″ W) (SJPO);
(7) Estancia San Nicolás, 22 Km SE San Miguel, Departamento San Miguel, Corrientes province (28° 07′35.03″ S, 57°25′53.73″ W) (SNIC);
(8) Estancia Santa Inés, RP 105, Km 8.5, Departamento Capital, Misiones province (27°31′59″ S, 55°52′22.03″ W) (SINE);
(9) Parque Provincial Piñalito, Departamento San Pedro, Misiones province (26°25′40.07″ S, 53°50′38.26″ W) (PPPI).

**Table 1:** Values of Prevalence (P), Mean Intensity (MI), total number of worms (TNW) and Relative Dominance (RD) for *Hassalstrongylus dollfusi*, separated by host and locality. Numbers in parentheses indicate the total number of intestinal heligmonellids in the respective component communities. (GUAY) Estación de Animales Silvestres Guaycolec, Formosa province; (SRDO) Selvas del Río de Oro, Chaco province; (PULP) Puerto Las Palmas, Chaco province; (CAMP) Campus “Deodoro Roca”-Universidad Nacional del Nordeste, Corrientes province; (SCAY) Parque Provincial San Cayetano, Corrientes province; (SJPO) Estancia San Juan Poriahú, Corrientes province; (SNIC) Estancia San Nicolás, Corrientes province; (SINE) Estancia Santa Inés, Misiones province; (PPPI) Parque Provincial Piñalito, Misiones province.

		*Akodon azarae*	*Holochilus chacarius*	*Oligoryzomys flavescens*	*O. fornesi*	*O. nigripes*
GUAY	Host *n*	14	2	0	6	27
	*P* (%)	0	50	**–**	100	66.7
	MI	0	2	**–**	169.2	8.9
	TNW	0 (539)	2 (39)	**–**	1015 (1066)	160 (541)
	RD (%)	0	5.1	**–**	95.2	29.6
SRDO	Host *n*	1	29	0	7	0
	*P* (%)	0	20.7	**–**	100	**–**
	MI	0	2.2	**–**	12.7	**–**
	TNW	0 (158)	13 (3316)	**–**	89 (139)	**–**
	RD (%)	0	0.4	**–**	64	**–**
PULP	Host *n*	2	4	0	1	11
	*P* (%)	0	25	**–**	100	27.3
	MI	0	1	**–**	5	3.3
	TNW	0 (83)	1 (357)	**–**	5 (16)	10 (189)
	RD (%)	0	0.3	**–**	31.2	5.3
CAMP	Host *n*	0	1	13	0	0
	*P* (%)	**–**	0	92.3	**–**	**–**
	MI	**–**	0	101.5	**–**	**–**
	TNW	**–**	0 (74)	1218 (1218)	**–**	**–**
	RD (%)	**–**	0	100	**–**	**–**
SCAY	Host *n*	9	1	4	0	3
	*P* (%)	0	0	50	**–**	33.3
	MI	0	0	23	**–**	238
	TNW	0 (248)	0 (77)	46 (46)	**–**	238 (240)
	RD (%)	0	0	100	**–**	99.2
SJPO	Host *n*	2	0	3	0	5
	*P* (%)	50	**–**	100	**–**	40
	MI	303	**–**	42.7	**–**	2
	TNW	303 (303)	**–**	128 (133)	**–**	4 (29)
	RD (%)	100	**–**	96.2	**–**	13.8
SNIC	Host *n*	0	0	6	0	16
	*P* (%)	**–**	**–**	100	**–**	75
	MI	**–**	**–**	36.5	**–**	7.8
	TNW	**–**	**–**	219 (219)	**–**	93 (93)
	RD (%)	**–**	**–**	100	**–**	100
SINE	Host *n*	0	0	4	0	3
	*P* (%)	–	–	75	–	0
	MI	–	–	12.7	–	0
	TNW	–	–	38 (38)	–	0 (8)
	RD (%)	–	–	100	–	0
PPPI	Host *n*	0	0	1	0	17
	*P* (%)	–	–	100	–	0
	MI	–	–	2	–	0
	TNW	–	–	2 (23)	–	0 (499)
	RD (%)	–	-	8.7	–	0
Total	Host *n*	28	37	31	14	82
	*P* (%)	3.6	21.6	87.1	100	43.9
	MI	303	2	61.1	79.2	14
	TNW	303 (1331)	16 (3867)	1651 (1677)	1109 (1221)	505 (1361)
	RD (%)	22.8	0.4	98.5	90.8	37.1

*Prevalence and mean intensity:* see Table 1.

*Material studied and deposited:* see Table 2

**Table 2:** Examined material of *Hassalstrongylus dollfusi*. Acronyms for localities: IPAF: Instituto de Investigación para la Pequeña Agricultura Familiar del Noreste Argentino; GUAY: Estación de Animales Silvestres Guaycolec; SRDO: Selvas del Río de Oro; PULP: Puerto Las Palmas; CAMP: Campus “Deodoro Roca”-Universidad Nacional del Nordeste; SCAY: Parque Provincial San Cayetano; SJPO: Estancia San Juan Poriahú; SNIC: Estancia San Nicolás; SINE: Estancia Santa Inés; PPPI: Parque Provincial Piñalito. Acronyms for collections: CNP: Centro Nacional Patagónico – CCT – CONICET (Mammalogical Collection), Puerto Madryn, Argentina; MLP: Museo de La Plata (Mammalogical Collection), La Plata, Argentina; MLP-He: Museo de La Plata (Helminthological Collection), La Plata, Argentina.

Province	Locality	Lat (S)	Long (W)	Year	Host species	Host collection No.	Host field No.	Infrapopulation	Nematodes collection No.
Formosa	IPAF	25°12′09.91″	58°07′13.71″	2008	*Holochilus chacarius*		LTU 561	1♂	MLP-He 7746 (1♂)
						CNP 2391	LTU 565	1♂	
						CNP 3943	LTU 571	1♂	MLP-He 7746 (1♂)
	GUAY	25°58′57.08″	58°10′04.00″	2012	*Oligoryzomys nigripes*		CG 83	3♂, 1♀	MLP-HE 7748 (2♂, 1♀)
							CG 93	1♀	
							CG 96	1♂	MLP-HE 7748 (1♂)
							CG 118	1♀	
							CG 125	1♀	
						CNP 3877	CG 133	1♀	
				2013	*H. chacarius*	CNP 4775	CG 448	2♂	
					*O. fornesi*	CNP 5271	CG 435	1♂, 1♀	
						CNP 5010	CG 444	25♂, 22♀	
							CG 445	35♂, 42♀	
							CG 446	85♂, 105♀	MLP-He 7753 (6♂, 7♀)
						CNP 5277	CG 449	230♂, 243♀	
							CG 453	101♂, 125♀	MLP-He 7753 (1♀)
					*O. nigripes*		CG 372	1♀	
						CNP 4819	CG 416	2♂	
						CNP 4736	CG 418	5♂, 3♀	
							CG 420	1♂	
							CG 421	32♂, 61♀	
						CNP 5073	CG 422	4♂, 1♀	
						CNP 5259	CG 424	1♂, 2♀	
							CG 426	4♂, 10♀	MLP-HE 7748 (4♂, 4♀)
							CG 427	3♂, 3♀	
							CG 428	1♂, 2♀	
							CG 438	1♂, 10♀	
						CNP 5058	CG 451	4♂, 1♀	
Chaco	SRDO	26°47′23.35″	58°57′37.81″	2000	*H. chacarius*		RORO 002	1♂	MLP-He 7747 (1♂)
						CNP 3956	RORO 023	3♂	MLP-He 7747 (1♂)
							RORO 033	3♂	MLP-He 7747 (2♂)
						CNP 3954	RORO 035	1♂	MLP-He pending (1♂)
						CNP 3953	RORO 056	1♂	
							RORO 058	3♂	MLP-He 7747 (3♂)
							RORO 060	2♂	MLP-He 7747 (2♂)
				2013	*O. fornesi*		CG 404	2♂	MLP-He 7754 (2♂)
							CG 405	8♂, 9♀	MLP-He 7754 (2♂, 2♀)
							CG 408	5♂, 2♀	
							CG 409	26♂, 23♀	MLP-He 7754 (5♂, 3♀)
							CG 410	5♂, 7♀	MLP-He 7754 (1♂)
							CG 412	1♀	
							CG 413	1♂	
	PULP	27°04′45.00″	58°40′06.30″	2008	*O. fornesi*		LTU 585	2♂, 3♀	
	7 km S PULP	27°09′40.51″	58°40′27.29″	2008	*O. nigripes*	CNP 1748	LTU 589	1♀	
						CNP 5635	LTU 593	2♂, 4♀	MLP-He 7749 (1♂, 3♀)
						CNP 5723	LTU 595	2♂, 1♀	MLP-He 7749 (2♂, 1♀)
	2,5 km NW PULP	27°04′45.00″	58°40′06.30″	2008	*H. chacarius*	CNP 3937	LTU 605	1♀	MLP-He 7759 (1♀)
Corrientes	CAMP	27°28′09.17″	58°49′50.29″	2013	*O. flavescens*		RO-197	75	
							RO-211	32	
							RO-216	39	
							RO-218	39	
							RO-219	17	
							RO-221	22	
				2014			RO-347	108	
							RO-348	65	
							RO-352	230	
							RO-355	233	
							RO-358	301	
							RO-363	57	
	SCAY 1	27°32′43.88″	58°40′33.73″	2007	*O. flavescens*	CNP 5617	LTU 423	21♂, 20♀	MLP-He 7755 (1♂, 1♀)
							LTU 440	3♂, 2♀	
	SCAY 2	27°34′15″	58°41′41″	2011	*O. nigripes*		RO-022	238	MLP-He 7750 (6♂, 6♀)
	SJPO	27°42′34.09″	57°11′19.08″	2003	*Akodon azarae*	MLP 18.III.02.11	211	127♂, 176♀	MLP-He 7758 (4♂, 7♀)
					*O. flavescens*	MLP 17.XII.01.11	193	19♂, 17♀	MLP-He 7756 (3♂, 4♀)
					*O. nigripes*	MLP 27.XII.01.8	209	1♂, 2♀	MLP-He 7751 (1♂, 1♀)
						MLP 27.XII.01.9	213	1♂	MLP-He 7751 (1♂)
				2007	*O. flavescens*		LTU 413	23♂, 37♀	
							LTU 416	14♂, 18♀	
	SNIC	28°07′35.03″	57°25′53.73″	2010	*O. flavescens*	CNP 5606	UP 1070	1♂, 2♀	
							UP 1083	32♂, 33♀	
							UP 1095	31♂, 22♀	
							UP 1097	17♂, 28♀	
						CNP 5542	UP 1098	8♂, 16♀	
						CNP 5631	UP 1107	12♂, 14♀	
					*O. nigripes*		UP 1074	8♂, 10♀	MLP-He 7752 (1♂)
						CNP 5628	UP 1076	1♀	
							UP 1077	18♂, 11♀	
							UP 1080	1♂, 4♀	MLP-He 7752 (1♀)
						CNP 5640	UP 1081	3♂, 1♀	
							UP 1088	1♂, 4♀	
						CNP 5603	UP 1090	3♀	
							UP 1091	1♂, 4♀	
							UP 1100	3♂, 4♀	
						CNP 5622	UP 1101	4♂, 3♀	MLP-He 7752 (2♂, 2♀)
						CNP 5660	UP 1102	3♂, 1♀	MLP-He 7752 (1♂, 1♀)
						CNP 5611	UP 1103	1♂, 6♀	
Misiones	SINE	27°31′59″	55°52′22.03″	2009	*O. flavescens*		LTU 697	13♂, 22♀	MLP-He 7757 (5♂, 6♀)
				2018	*O. flavescens*		CG 751	2♂	
							CG 775	1♀	
	PPPI	26°25′40.07″	53°50′38.26″	2018	*O. flavescens*		CG 805	2♀	

#### Redescription (Figs. 2–6, Table 3)

**Figure 2:**
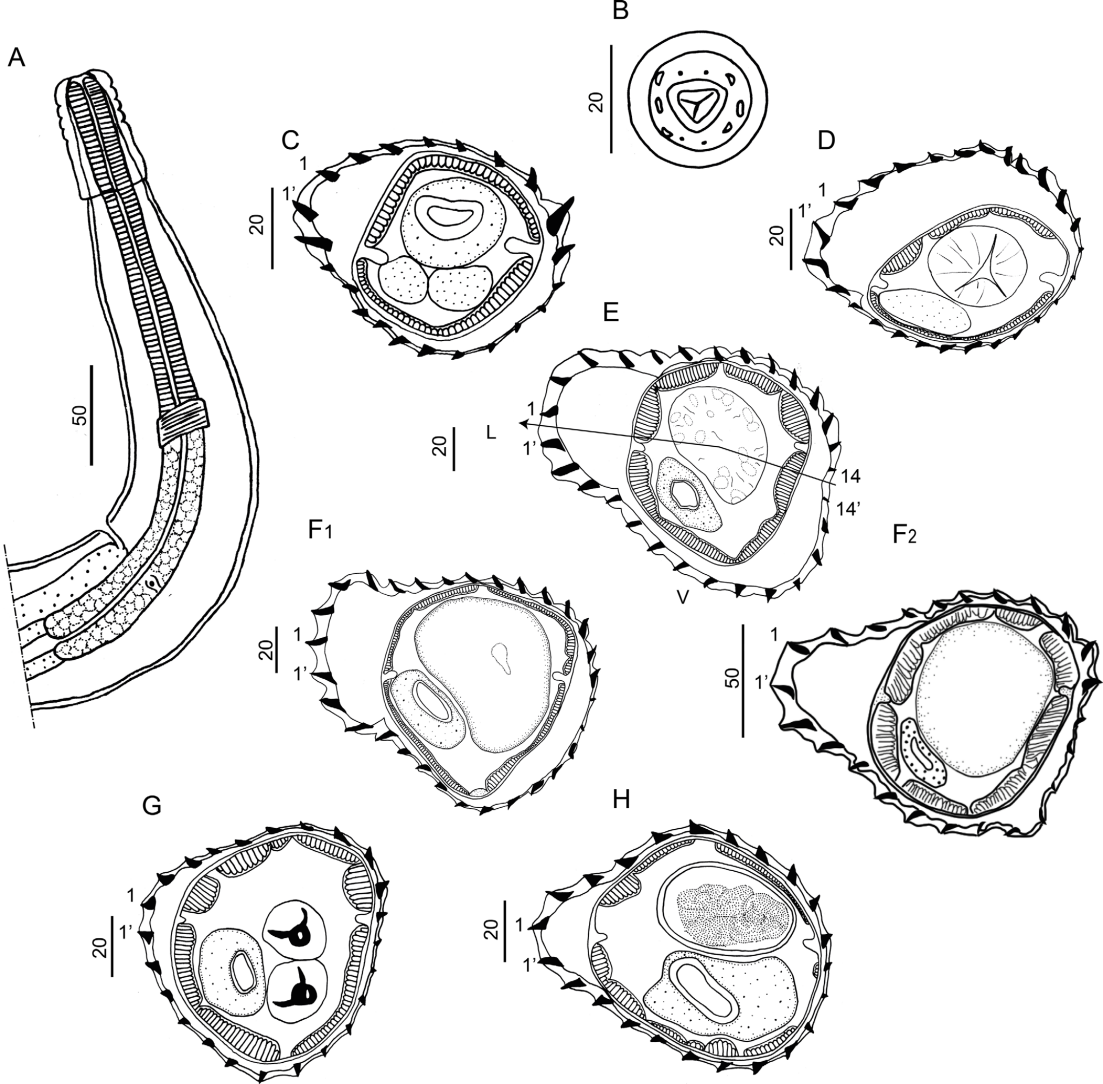
*Hassalstrongylus dollfusi*. A, male, anterior extremity, left lateral view. B, female, head, apical view. C–H, synlophe in transverse sections of the body: C, D, at oesophago-intestinal junction, C, male, D, female; E, F, at mid-body, E, male, F, female, (1) this work, (2) modified after Durette-Desset (1969); G, male, at level of spicules; H, female at level of uterus. Abbreviations: L, left, V, ventral. Arrow represents the axis of orientation of the ridges. All sections oriented as in E.

*General:* medium-sized nematodes, generally uncoiled, sometimes loosely coiled in 1–3 spirals. Cephalic vesicle present. Excretory pore always within posterior half of oesophagus (Table 3). Deirids at same level of excretory pore in most specimens (74% of 50 specimens), sometimes 3–25 µm anterior or posterior to it (Fig. 2A).

**Table 3:** Comparative measurements of males and females of *Hassalstrongylus dollfusi* from the murid type host (from Díaz-Ungría 1963) and five native cricetid hosts (this work).

	*Mus musculus*		*Akodon azarae*		*Holochilus chacarius*		*Oligoryzomys flavescens*		*O. fornesi*		*O. nigripes*	
	Díaz-Ungría 1963		This work		This work		This work		This work		This work	
	♂ (*n* = np)	♀ (*n* = np)	♂ (*n* = 10)	♀ (*n* = 7)	♂ (*n* = 14)	♀ (*n* = 1)	♂ (*n* = 9)	♀ (*n* = 10)	♂ (*n* = 14)	♀ (*n* = 12)	♂ (*n* = 21)	♀ (*n* = 20)
Body length (mm)	3.53	2.84–6.56	2.5–4.6 (3.2)	3.1–6.2 (4.4)	3.1–5.8 (3.9)	4.4	3.9–5.2 (4.5)	5.1–9.5 (6.8)	3.5–5.4 (4.4)	3.7–7.9 (6.8)	2.3–6 (4.4)	4.7–8.1 (6.5)
Body width	140	np	70–130 (91)	73–127 (91)	90–200 (113)	100	105–138 (117)	105–176 (131)	90–160 (116)	80–175 (127)	65–140 (120)	85–190 (133)
Cephalic vesicle length	65–70	53–75	35–63 (44)	40–60 (50)	48–70 (58)	40	48–70 (58)	60–70 (65)	53–75 (65)	50–75 (65.5)	40–68 (60)	48–76 (61) (*n* = 14)
Cephalic vesicle width	33–35	28–38	24–31 (27)	23–38 (31)	25–45 (35)	32	28–45 (34)	32–40 (36)	27–40 (33.5)	28–46 (38)	28–40 (34)	28–45 (36) (*n* = 15)
Oesophagus length	310	300–360	218–353 (263)	270–385 (314)	260–350 (316)	310	270–330 (302)	275–405 (351)	265–357 (326.5)	260–390 (341)	270–355 (322)	280–408 (344)
Nerve ring *	np	np	95–157 (115)	117–150 (130)	120–185 (151) (*n* = 9)	100	98–200 (140)	103–200 (154) (*n* = 7)	115–190 (154)	120–170 (152) (*n* = 7)	100–180 (160) (*n* = 14)	120–193 (153) (*n* = 12)
Excretory pore *	250	180–190	115–320 (175)	160–220 (179) (*n* = 5)	172–340 (246)	no	148–305 (221)	165–301 (239) (*n* = 8)	185–307 (234) (*n* = 10)	200–268 (239) (*n* = 5)	190–290 (254) (*n* = 12)	220–320 (257) (*n* = 12)
EP/OeL (%)	np	np	52.8–90.7 (65)	57.1–65.9 (61.4) (*n* = 5)	61.4–101.5 (77.7)	no	54.2–94.1 (72)	53.6–83.6 (67) (*n* = 8)	60.6–79 (70.1)	58.8–73.9 (66.1) (*n* = 5)	61.3–85.7 (77.5) (*n* = 12)	64.1–86.1 (74) (*n* = 12)
Deirids *	np	np	138–235 (170) (*n* = 4)	160–225 (189)	172–340 (249)	no	148–240 (185) (*n* = 4)	220–298 (259) (*n* = 5)	235–327 (267) (*n* = 4)	200–268 (233) (*n* = 3)	190–290 (253) (*n* = 9)	225–320 (265) (*n* = 10)
Spicules length	625–660	–	460–655 (547)	–	530–680 (585)	–	600–710 (646)	–	575–700 (656)	–	550–715 (652)	–
SpL/BL (%)	np	–	13.6–20.8 (17)	–	11.7–18.1 (15)	–	13.1–15.7 (14)	–	12.5–19.6 (15.2)	–	10.6–24.4 (15.5)	–
Genital cone length	np	–	29–40 (33)	–	28–45 (36) (*n* = 12)	–	35–46 (38) (*n* = 5)	–	30–50 (37.9) (*n* = 7)	–	35–65 (43.9) (*n* = 8)	–
Gubernaculum length	np	–	20–30 (25) (*n* = 5)	–	10–25 (20) (*n* = 5)	–	20–28 (23) (*n* = 3)	–	18–25 (22) (*n* = 5)	–	18–25 (22.5) (*n* = 6)	–
Vulva **	–	200–215	–	140–220 (164)	–	150	–	138–253 (186)	–	110–252 (196)	–	120–236 (181)
Vagina vera	–	np	–	20–60 (35)	–	16	–	25–43 (33) (*n* = 6)	–	20–50 (37) (*n* = 8)	–	10–41 (25)
Vestibule length	–	np	–	60–105 (78) (*n* = 5)	–	100	–	97–135 (119)	–	90–145 (120)	–	105–150 (123)
Sphincter length × width	–	np	–	27–35 (30) × 34–45 (39)	–	30 × 30	–	29–38 (33) × 31–46 (40)	–	20–40 (32) × 28–44 (39)	–	25–41 (39) × 32–46 (39)
Infundibulum length	–	np	–	110–168 (142) (*n* = 5)	–	100	–	124–185 (167) (*n* = 5)	–	100–190 (145) (*n* = 2)	–	100–182 (131) (*n* = 13)
Uterus length	–	np	–	395–1050 (661)	–	380	–	830–1600 (1218)	–	735–1120 (946) (*n* = 9)	–	510–1560 (1119)
UtL/BL (%)	–	np	–	12.9–18.8 (15.6)	–	8.7	–	14.4–21.1 (17)	–	12–19.3 (14.6) (*n* = 9)	–	9.4–24.2 (17)
Tail length	–	50–80	–	40–60 (47)	–	50	–	50–85 (60)	–	45–85 (58)	–	40–65 (51)
Number of eggs	–	np	–	8–25 (13)	–	16	–	16–80 (46)	–	11–61 (30.5)	–	6–69 (31)
Eggs length × width	–	50–60 × 29–35	–	55–70 (61) × 35–40 (39) (*n* = 5)	–	no	–	50–69 (63) × 30–44 (36)	–	40–66 (57) × 26–43 (32)	–	40–65 (53) × 27-40 (33)

Abbreviations: no = not observed, np = not provided.

* Distance to apex.

** Distance to posterior extremity.

*Head:* in apical view, triangular buccal opening surrounded by thick ring; 2 amphids, 4 externo-labial (2 dorsal, 2 ventral), and 4 cephalic papillae visible; lateral externo-labial papillae probably fused with amphids (Fig. 2B).

**Figure 3:**
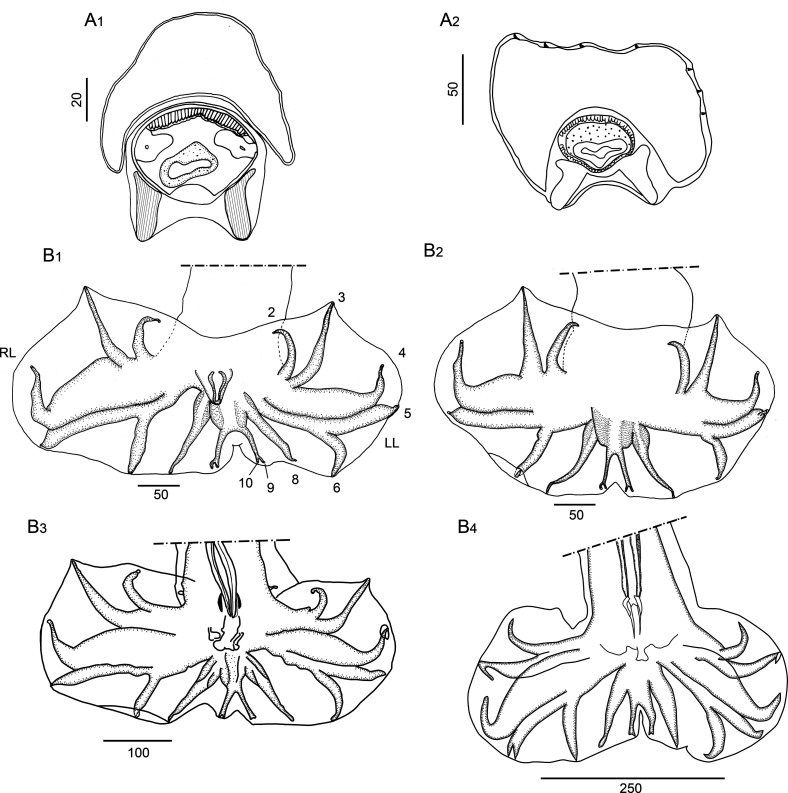
*Hassalstrongylus dollfusi*. A, female, postvulvar alae in transverse section of the body: (1) this work, (2) modified after Durette-Desset (1969). B, male, bursa, ventral views, (1, 2) this work, (1) with distal divergence of left ray 6, (2) with proximal divergence of left ray 6; (3) modified after Durette-Desset (1969), (4) modified after Díaz-Ungría (1963). Abbreviations: RL, right lobe, LL, left lobe.

*Synlophe* (studied in 6 males and 6 females): in both sexes, cuticle bearing longitudinal, uninterrupted ridges with well-developed struts. Ridges appearing gradually mainly on left side just posterior to cephalic vesicle up to oesophago-intestinal junction; disappearing just anterior to bursa in male and reaching distal cuticular inflation in females. Number of ridges variable in proportion to body diameter: at oesophago-intestinal junction, 22-26 in both sexes (Figs. 2C, 2D); at midbody 22–31 in males, 22–30 in females (Figs. 2E, 2F); within posterior third of body length 24–26 in males, 23–28 in females (Figs. 2G, 2H). All ridges medium sized, except those on right ventral quadrant (smallest) and those associated with lateral hypodermal chords (slightly larger). At oesophago-intestinal junction, especially in male, difference in ridge size more marked than at midbody. At midbody, ridge 2′ largest. All ridges well oriented, determining a double axis of orientation of the ridges in both sexes: left axis inclined at 83°–84° with respect to sagittal axis, right axis at 70°–72°. Within distal third of body length: in both sexes, similar number and orientation than at midbody, differences in ridge size less marked (Figs. 2G, 2H). In females, between vulva and anus, presence of paired ventral postvulvar alae supported by hypertrophied struts (Fig. 3A).

**Figure 4:**
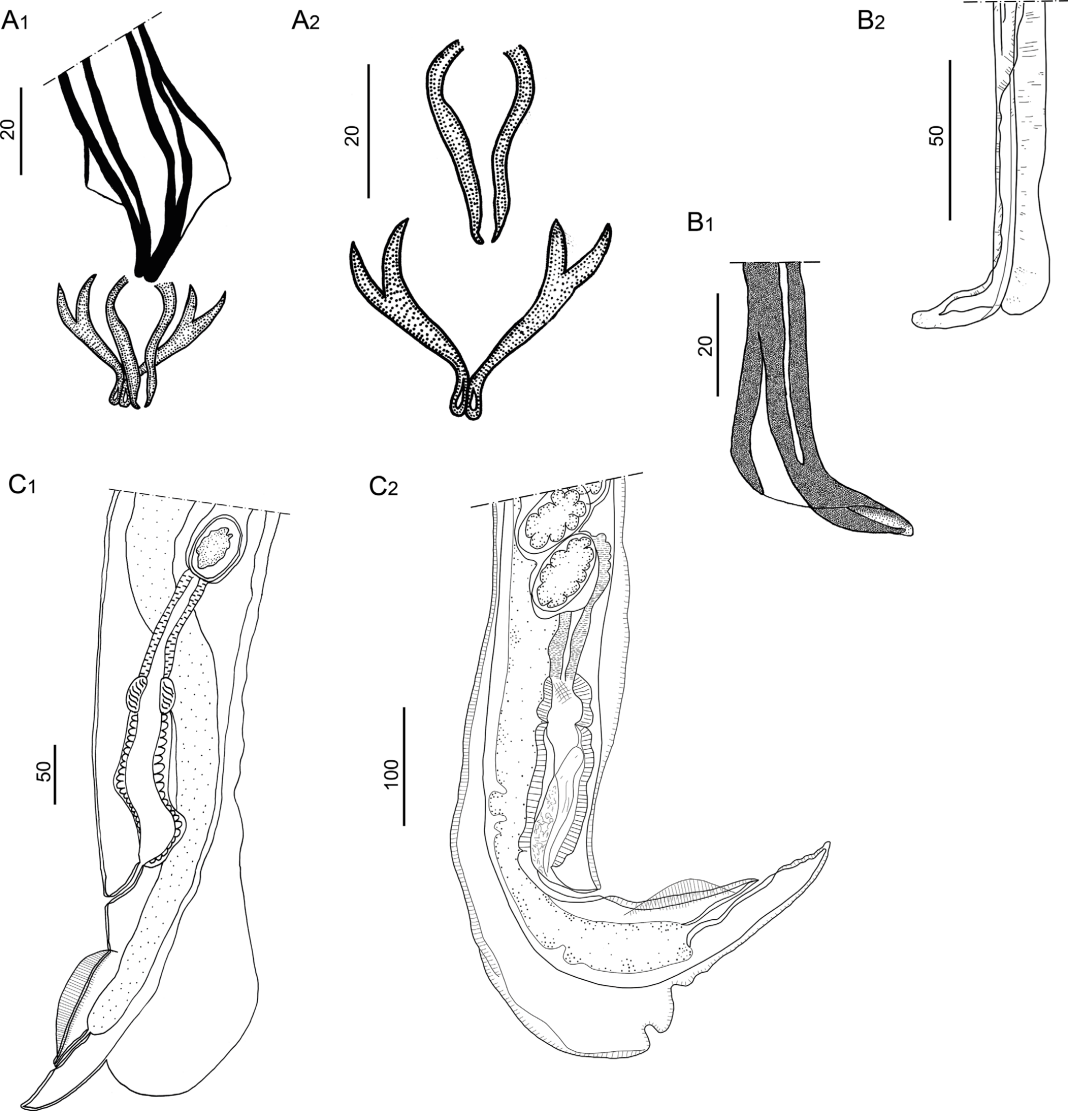
*Hassalstrongylus dollfusi*. A, B male. A, telamon: (1) ventral view with spicules tips, (2) ventral view, pieces separated. B, tip of spicule: (1) this work, (2) modified after Durette-Desset (1969). C, female posterior extremity: (1) this work, left lateral view, (2) modified after Durette-Desset (1969), right lateral view.

**Figure 5:**
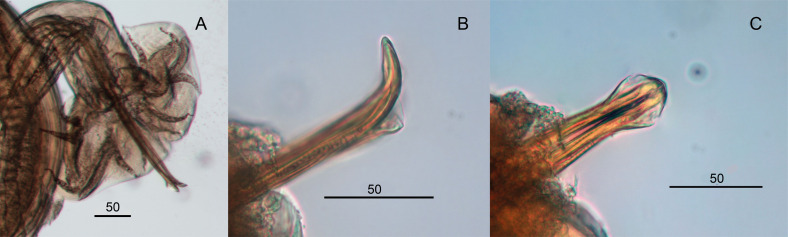
*Hassalstrongylus dollfusi*. Male. A, bursa, ventral view; B, C, tips of spicules, B, right lateral view, C, ventral view.

*Males* (measurements reported by host in Table 3): bursa slightly dissymmetrical, with right lobe larger than left (Figs. 3B, 5A). Prebursal papillae not observed. Right lobe with pattern of type 2-2-1 tending to 1-3-1. Left lobe with pattern of type 2-3 tending to 2-2-1. Rays 2 shorter than rays 3 and curved toward median line. Rays 4 and 5 diverging at extremity, rays 4 longer, strongly curved anteriad, rays 5 straight. Right ray 4 markedly thicker than left one. Right ray 6 diverging from common trunk of rays 2–6 between rays 2 and 3; left ray 6 diverging distally in different degrees to ray 3: from just distal (Fig. 3B2) up to halfway between ray 3 and divergence of rays 4 and 5 (Fig. 3B1). Rays 8 thickened at base, arising symmetrically from base of dorsal ray. Dorsal ray short, thickened at base, dividing within middle third into two branches, each one bifurcated distally into two papillae, external rays 9 and internal rays 10. Genital cone well developed. Telamon complex, composed of several sclerotized pieces: two dorsal simple branches forming a pincer or caliper, and two ventral bifid branches articulated distally (Fig. 4A). Gubernaculum inconspicuous, Spicules thin, subequal, alate. Each spicule tip spatulate or spoon-shaped, bent at right angle with respect to main axis of spicule. Just before bending, spicular ala enlarged and ending widely, with the appearance of a heeled shoe (Figs. 4B and 5B, 5C).

**Figure 6:**
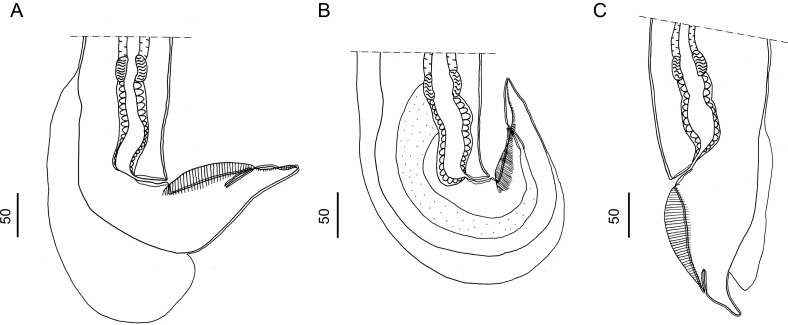
*Hassalstrongylus dollfusi*. Female, posterior extremities. A, curved ventrally at right angle, cuticular inflation ends abruptly, almost perpendicular (most specimens); B, strongly curved ventrally, cuticular inflation gradually tapering, ends obliquely; C, straight, cuticular inflation less developed. A, B, right lateral views; C, left lateral view.

*Females* (measurements reported by host in Table 3): reproductive tract monodelphic. Uterus less than 20% of BL, number of eggs variable (see Table 3). Infundibulum slightly longer than vestibule. Distal end, in most specimens, curved ventrally to different degrees, more rarely straight (on 50 females examined, 19% strongly curved, 30% curved at right angle, 28% slightly curved and 23% straight). Cuticle inflated to different degrees from level of sphincter or vestibule up to halfway between vulva and anus (Figs. 4C and 6). Two latero-ventral alae present between vulva and anus (Fig. 4C). Tail conical, not invaginated (Figs. 4C and 6).

#### Diagnosis

Diagnostic characters of *H. dollfusi* males are: the morphology of the caudal bursa (particularly hypertrophy of right ray 4 and thickening of dorsal ray and rays 8 at their base), and the peculiar shape of the distal tip of the spicules. Diagnostic characters of the female are: the dorsal cuticular inflation at ovejector level and the subventral postvulvar alae supported by hypertrophied struts. These characters were present in all specimens studied herein and, compared to those in the original description [12] and further redescription [15], did not show any differences. In addition, the synlophes at midbody of males and females examined were congruent with those described by Durette-Desset [15]. Related to the measurements, they were homogenous among specimens from the different host species, including the type material from *M. musculus*. The specimens parasitizing *A. azarae* and *H. chacarius* were slightly smaller (Table 3).

Slight variability with respect to the original description was found only in two characters: the level of divergence of left ray 6 of the bursa (more distal in three of our males due to a larger right lobe, see Fig. 3B1), and the general aspect of the female posterior end. Related to the latter, our specimens showed, independently of the host species, different degrees of curvature and different degrees of inflation of the cuticle (Fig. 6); whereas in the type material the posterior end is curved at a right angle and the cuticular inflation ends abruptly, almost perpendicular to the body wall (Fig. 4C2). A degree of variability in these types of qualitative traits is expected when a large number of specimens are examined. Consequently, we identified our specimens as *H. dollfusi*.

Recently, Gómez-Muñoz et al. [24] reported, in two species of *Oligoryzomys* Bangs from Corrientes province, Argentina, specimens of nippostrongylines which were identified provisionally as *Stilestrongylus* sp., mainly based on characters of the synlophe. However, a more detailed study on those specimens indicated that they should be attributed to *H. dollfusi* and consequently they are included in this work.

Values of prevalence, mean intensity and relative dominance of *H. dollfusi* (Table 1) show a strong preference of the parasite by species of *Oligoryzomys,* with the highest prevalence and mean intensity in *O. fornesi* (global *P* = 100%, MI = 79.2) followed by *O. flavescens* (*P* = 87.1%, MI = 61.1) and *O. nigripes* (*P* = 43.9%, MI = 14). In contrast, in *H. chacarius*, the prevalence was lower (*P* = 21.6%), and the intensity extremely low (MI = 2). In this host, the intensity of infection never exceeded 3 worms, and only one female worm was found in the whole sampling (Tables 1–3). In *A. azarae*, *H. dollfusi* was found in only one out of 28 examined hosts from the localities reported in this paper (*P* = 3.6%) (Fig. 1; Table 1). The highest values of relative dominance of *H. dollfusi* in the community of intestinal nippostrongylines were registered in *O. flavescens* and *O. fornesi*, with 98.5 and 90.8 respectively, followed by *O. nigripes* (with 37.1) and the unique *A. azarae* with 22.8 (Table 1).

## Discussion

The finding of *H. dollfusi* in ten different localities in an area of about 19,000 km^2^, in five different host species and through several sampling events (see Table 2 for sampling details) allows us to consider *H. dollfusi* a usual component of the helminth fauna of sigmodontine rodents in this area. However, the values of prevalence and mean intensity, when considered globally by host species, greatly varied, being higher in the three species of *Oligoryzomys* and remarkably lower in *H. chacarius* and *A. azarae* (Table 1).

Data in Table 1 indicate that in the localities surveyed *O. flavescens*, *O. fornesi*, and *O. nigripes* act as primary hosts of *H. dollfusi*. The presence of the parasite in *H. chacarius* and *A. azarae* is, instead, less frequent, occurring when these latter share the habitat with the primary hosts; and even in such cases, *H. dollfusi* never appears as an important component of the helminth community. It is worth noting that in the localities reported herein, many other species of sigmodontine hosts were captured and examined for helminths. However, none of them was found to harbour *H. dollfusi*. These hosts were: *Akodon montensis*, *Calomys callidus*, *C. callosus, Necromys lasiurus*, *N. obscurus*, *Oxymycyterus rufus* and *Sooretamys angouya*.

As stated above, *H. dollfusi* was, up to now, only known from its type host *Mus musculus*, a non-native rodent. Díaz-Ungría [12] remarked that the host of *H. dollfusi* was captured in the wild (forest). In this sense, it is worth noting that the type locality (Tiara, Aragua Department, Venezuela) at the time of the study was a mining settlement (R. Guerrero, pers. comm.). Therefore, the presence of mice should not be surprising, and it is not unexpected that mice became infected with autochthonous nippostrongylines during incursions in the forest in the vicinities of the locality. Durette-Desset [15], when redescribing *H. dollfusi*, remarked that it was unlikely that the mouse was the primary host of the parasite, and that this latter should be found among the autochthonous cricetids. The fact that *H. dollfusi* was never reported again from mice supports this hypothesis. On the other hand, the lack of subsequent records of the parasite even from native hosts should be attributed probably to both the paucity of helminthological surveys and the host preference showed by the parasite.

The primary host of *H. dollfusi* should undoubtedly be an autochthonous cricetid, but which one remains a question. In South America, the genus *Oligoryzomys* has 19 species distributed in a continuum from north Colombia and Venezuela to extreme southern Chile and Argentina (for a detailed distribution of the *Oligoryzomys* species see [42]). The specimens of *H. dollfusi* studied herein, compared to those from the type locality, show remarkable morphological homogeneity. Therefore, even when the present finding is far from the type locality of *H. dollfusi*, it would not be unreasonable to suppose that the parasite could be present in an area comprising at least from the type locality in Venezuela south to northern Corrientes province in Argentina, parasitizing different species of *Oligoryzomys*.

However, it is also worth noting the absence of *H. dollfusi* in several other localities where populations of *Oligoryzomys* spp. were present and abundant. This was observed in our study (Fig. 1), but also in numerous studies carried out by Argentinian and Brazilian researchers regarding the parasitic fauna of different species of *Oligoryzomys* [6–8, 28, 31, 33, 38, 39].

Indeed, *H. dollfusi* was not found in localities south of 28° S (this work) where *O. flavescens* and *O. nigripes* were captured and examined for parasites (Fig. 1, localities F and H). Nor was the parasite found during an extensive study on the helminth assemblage of sigmodontine rodents (included *O. flavescens* and *O. nigripes*) from a broad wetland area of the Río de la Plata, Buenos Aires province, Argentina between 34°45′ S, 58°06′ W and 34°56′ S, 57°42′ W [31].

In a similar way, *H. dollfusi* has never been reported in any of the numerous studies on helminths of sigmodontine rodents in different localities from the Atlantic Forest from Brazil [6–8, 28, 38, 39] or Argentina [33], even when *O. nigripes* was one of the rodent species most commonly captured in such localities.

The Trichostrongylina are characterized by direct life cycles, in which the first three larval stages are free in the external environment and the stage infectious to the definitive host is the third-stage larva [2, 22]. Therefore, the presence and persistence of the parasite in an area will be related both to the presence of populations of suitable definitive hosts and to the existence of adequate environmental conditions (temperature, humidity level, soil and vegetation type, etc.) for the free-living stages. Specific environmental requirements for the free-living stages of *H. dollfusi* are still unknown, but a probable constraint in these conditions may explain the absence of the parasite in well-surveyed populations of *Oligoryzomys* spp. south of 28° S in Argentina [31] and eastward in the Argentinean and Brazilian Atlantic Forest [6–8, 28, 33, 38, 39]. The extremely low value of RD of *H. dollfusi* in the unique positive locality corresponding to the Atlantic Forest biome (PPPI, Fig. 1, Table 1) is consistent with this hypothesis.

A significant sampling effort and thorough taxonomical work in the extensive intermediate area between the type locality and the area surveyed herein would greatly improve our knowledge of hosts and geographical distribution of *H. dollfusi*. Meanwhile, this study provides the first identification and redescription of this species in southern South America, from autochthonous hosts, and more than 50 years after its original description.
